# Application Effect of MF-OP on Collection of Trivalent Holmium from Rare Earth Mining Wastewater

**DOI:** 10.3390/ijerph20021498

**Published:** 2023-01-13

**Authors:** Liang Pei, Liying Sun

**Affiliations:** 1 National Engineering Technology Research Center for Desert-Oasis Ecological Construction, Xinjiang Institute of Ecology and Geography, Chinese Academy of Sciences, Urumqi 830011, China; 2 Xinjiang Key Laboratory of Environmental Pollution and Bioremediation, Xinjiang Institute of Ecology and Geography, Chinese Academy of Sciences, Urumqi 830011, China; 3 Institute of Geographic Sciences and Natural Resources Research, Chinese Academy of Sciences, Beijing 100101, China; 4 University of Chinese Academy of Sciences, Beijing 100049, China

**Keywords:** microtube microfilter with organic phosphoric acid, trivalent holmium, wastewater portion, enriched portion, collection proportion

## Abstract

Microtube microfilter with organic phosphoric acid (expressed as MF-OP) containing a wastewater portion with buffer fluid and an enriched portion with nitric acid fluid and organic phosphoric extractant dissolved in benzin has been studied for its trivalent holmium (expressed as Ho(III) collection from rare earth wastewater. Common parameters affecting the collection effect have been investigated, including hydrogen ion molar concentration (molar concentration can be expressed as *Cm*) or pH value, initial concentration (expressed as *Co*) of Ho(III), ion-force of rare earth wastewater, voluminal proportion of organic phosphoric extractant with benzin and nitric acid fluid (expressed as *Vr*), nitric acid *Cm*, extractant *Cm*, and type of acid fluid in an enriched portion. The virtues of MF-OP compared to the traditional collection was explored. The impacts of hydrodynamic characteristics (steadiness and current speed) and MF parameter factors (inradius of tube, tube–shell thickness, proportion of holes) on the collection performance of MF-OP for Ho(III) collection were also considered. The test results displayed that the greatest collection conditions of Ho(III) were attained as nitric acid *Cm* was 4.00 mol/L, extractant *Cm* was 0.220 mol/L, and *Vr* was 0.8 in the enriched portion, and pH value was 4.60 in the wastewater portion. Ion- force of rare earth wastewater had no noticeable outcome on Ho(III) collection. The collection proportion of Ho(III) was attainable to 93.1% in 280 min, while *Co* was 1.80 × 10^−3^ mol/L.

## 1. Introduction

Trivalent rare earth metallic ions (expressed as Re(III)) have been widely used. They can be used alone in certain fields or together with other substances. Adding a certain amount of Re(III) or Re(III) compound in the metal manufacturing industry can improve the performance and quality of the alloy. Consequently, rare earth elements are called metallurgical vitamins in the metallurgical industry [1]. China has a large number of Re(III) minerals. We have a good opportunity to use these minerals, but how to obtain and collect Re(III) needs to be studied. At the same time, the efficient treatment technology of Re(III) wastewater is also a difficult problem in the current mining industry. At present, due to some domestic political reasons, we need to mine and excavate rare earth minerals, but some industrial wastewater that is difficult to treat will also be produced in the procedure of mining and production. In this study, Ho(III) is collected from the mining wastewater in a region. Whether we can extract useful Ho(III) while treating the wastewater cleanly is now an important research goal. A certain amount of Ho(III) containing wastewater has a certain negative impact on the human body and the natural environment. We detected a certain amount of Ho(III) in the plant roots around the ore, which indicates that the discharge of these wastewaters does not meet the national and industrial standards (<0.08 mg/L) [2]. Consequently, it is very likely that it has posed a threat to local human health. We need to treat these wastewaters containing Ho(III) and collect Ho(III). The existing traditional water treatment methods have a poor collection influence, so it is necessary to study new and efficient methods. On this basis, this study explored new methods for extracting and removing Ho(III), and provided theoretical support for the treatment of Ho wastewater in the future [3].

For the extraction and removal of heavy metals in wastewater, there are many traditional methods, and the comparison highlighted is the membrane method, where membrane reactor collection efficiency is high. Compared with conventional solvent collection methods, membrane reactors have the following advantages: collection has a short procedure, high speed, high enrichment, low consumption for regeneration, and low cost. In the early 1980s, an ion exchange membrane reactor system was used to extract Re(III) or common benzene in China, the support used various organophosphonates, and the Re(III) leaching liquid could be grouped, purified, and separated according to the need for posterior recycling [3]. In recent years, there have also been some scholars who have used stearic acid and phosphate as extractants to study the purification of rare earth elements Sm(III) from conventional membrane reactor systems and established collection models [4]. Some have also used composite membrane reactors to treat lanthanum in Re (III) wastewater [5]. Others have also established reaction systems of Re(III) element europium membranes with polypropylene porous membranes as support membranes and organophosphorus as support, and mathematical models of the membrane reaction systems and heavy metal ion collection procedures have been developed [6]. Relevant scholars at home and abroad [7] also studied the flat sandwich membrane reaction system, explored the technique to extract with parameters and influencing factors, compared the efficiency difference between different materials and thicknesses of membrane bodies in the collection procedure, and explored the collection rate and stability of the membrane reaction system and their interrelationships. These techniques provided the foundation for our study, but the removal efficiency and stability of these techniques remain to be improved.

The study of methods for fitting filtration devices with extractants is beginning to be initiated, but stability and efficiency are difficult to take into consideration simultaneously [8]. Many experts have also begun to investigate new structures to overcome previous defects to improve system stability and collection efficiency [9,10,11]. It has been suggested that microfiltration bodies as supporting elements, with the addition of various chemical reactions in combination with organophosphate liquids, can efficiently overcome the problem of microfiltration carrier leakage. Recently, some experts have also conducted corresponding research, and the research results show that the method is more efficient than previous methods [12,13,14,15]. Our research group has studied this field for many years, and based on previous techniques of dispersive supported fluid membrane reactor and hollow fiber membrane reactor [15], an MF-OP system with organic phosphoric acid in gasoline as the flow carrier is proposed [16,17].

At present, the application of MF-OP in the migration of Ho(III) has not been reported. Simultaneously, there are still some gaps in the mining wastewater treatment technology, and we will fill in the field of mining wastewater treatment using microtube microfilter for trivalent holmium. Aiming at the low efficiency of the traditional microtube microfilter method and the loss of organic solution, we have selected the MF-OP system for application and optimization. This paper has mainly explored and studied the feasibility of extracting Ho(III) using MF-OP. The migration of rare earth metallic ions was realized through membrane module design, carrier optimization, and migration percentage control. The migration process of rare earth metallic ions was studied, and a new method for extracting rare earth metallic ions using MF-OP was established. The study results can provide a scientific basis and new ideas for rare earth wastewater treatment in the mining industry.

## 2. Materials and Methods

### 2.1. MF-OP Collection Procedure

**Figure 1:**
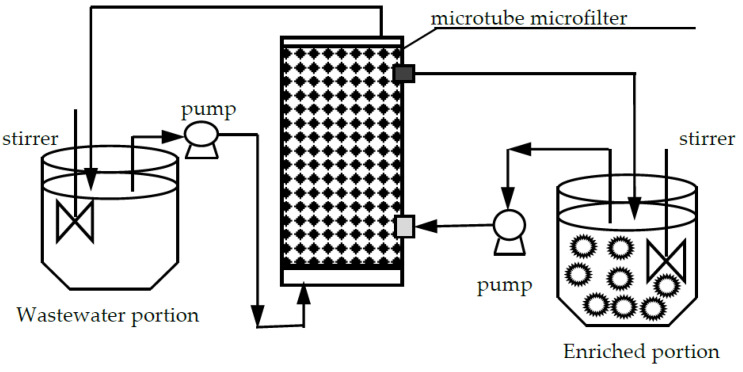
Installation of microtube microfilter reactor with organic phosphoric system procedure.

**Figure 2:**
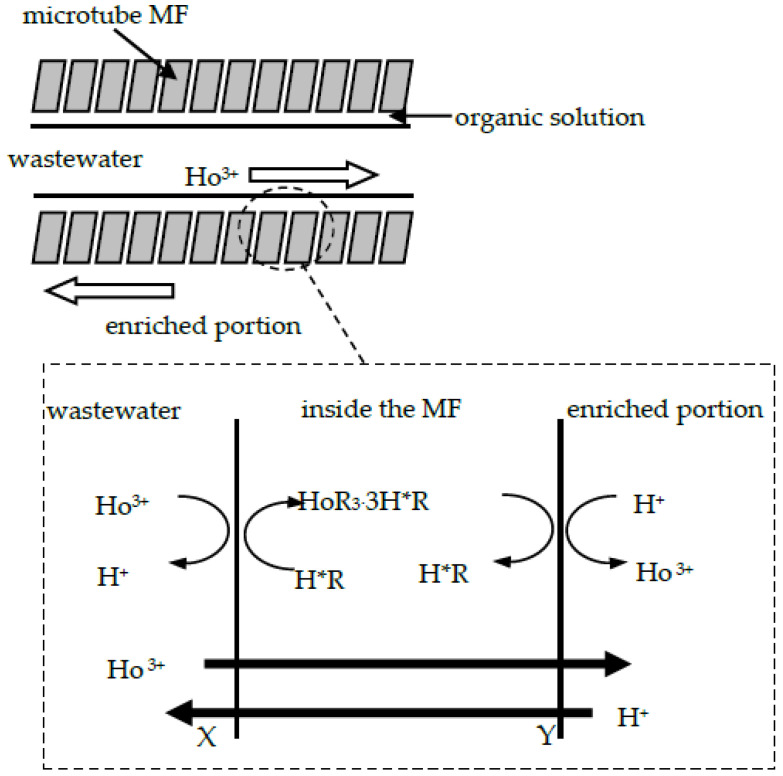
Diagrammatic sketch and description of Ho ^3+^ collection MF-OP.

MF-OP device is shown in Figure 1, and the theory of MF-OP practice was revealed in Figure 2, which designates the Re(III) collection procedure. The sewage section is prepared by dissolving a certain amount of Ho(CH_3_COO)_3_·4H_2_O in acetate buffer liquid with adjusted acidity value. The enrichment section uses organic phosphonate as the carrier and is dissolved in fuel oil. The extract is 3.0 mol/L aqua fortis liquid. The microtubule ultrafiltration body is soaked with organic liquid for at least 48 h to make the pores of the microtubule completely filled with organophosphorus liquid. In these experiments, there are two operation modes: (I) the extract enters the system through the outer wall of the ultrafiltration tube, and the sewage enters the system through the ultrafiltration tube; (II)the extract enters the system through the ultrafiltration tube, and the sewage enters the system through the outer wall of the ultrafiltration tube. Both fluids are transported in opposite directions and in a single path process. Generally speaking, due to the weak surface activity of the migration liquid, the use of a stirrer can mix the migration liquid with organophosphonic liquid and form droplets of the migration liquid in continuous organic liquid. That can be called the enriched section. During the experiment, there was a constant supply of organophosphonic liquid, that is, the enriched organophosphonic liquid was in contact with the micropores of MF. The continuous supply of this organophosphonic liquid ensures the stable and continuous operation of MF. In this way, the complex of rare earth metallic ion and carrier can diffuse to the interface between migration liquid and organic liquid in MF pores. Therefore, the direct contact between migration liquid and organophosphonic liquid provides effective mass transfer for rare earth metallic-ion migration. Secondly, once the migration of the target species is completed, the agitator used for migration will not work. The organic liquid suspension is allowed to separate the enrichment section into two sections: an organophosphonic liquid that is easy to wet the carrier hole and a migration liquid containing concentrated rare earth metallic ion. The concentrated extract is the product of this process. 

In order to prevent the leakage of organophosphonic liquid from the film hole, the pressure on both sides can be controlled through the flow velocity to make the pressure outside the ultrafiltration tube slightly positive and form a stable interface.

Based on our previous experiments, a stable Ho(III) molarity distribution was obtained in the sewage section and enrichment section after 22 min, and then a stable mass transfer performance was realized. Similar results were found when the enriched section flowed through the tube side. In later experiments, the initial stabilization time is set to more than 30 min to obtain more reliable results [17,18].

(a) Ho(III) diffuses from the sewage section to interface X.

(b) On the sewage side interface of MF-OP, Ho(III) is extracted from the sewage liquid with carrier organophosphorus (which can be (H ∗ R)_2_) in fuel oil, which can be expressed as [11]:

(1) $${\mathrm{Ho}}_{f}^{3+}+3{\left(H*R\right)}_{2,\mathrm{org}}\overset{K_{1}}{\underset{K_{-1}}{⇄}}\mathrm{Ho}R_{3}\cdot 3H*R+3H_{f}^{+}$$

where f and org represent sewage and organophosphonic liquid, respectively; (HR)_2_ shows that the organic phosphorus in fuel oil mainly exists in the form of dimer; *K*_1_ and *K*_−1_ represent the forward and reverse reaction percentage constants at the interface between the sewage section and the ultrafiltration microtubule.

(c) The metallic-ion complex (HoR_3_·3H ∗ R) was extracted by film X-Y.

(d) At the migration side interface of MF-OP, HoR_3_·3H ∗ R and metallic-ion Ho(III) dissolved in organophosphonic liquid are extracted by migration agent.

The migration reaction on the other side of the microtubule is as follows

(2) $$\mathrm{Ho}R_{3}\cdot 3H*R+3H_{s}^{+}\overset{K_{2}}{\underset{K_{-2}}{⇄}}{\mathrm{Ho}}^{3+}+3{\left(H*R\right)}_{2,\mathrm{org}}$$

where s represents the enrichment section; *K*_2_ and *K*_−2_ represent the forward and reverse reaction percentage constants at the interface between the organophosphonic liquid and enrichment section.

(e) The carrier organophosphorus returns from Y to X.

In this mechanism, the migration of Ho(III) by MF-OP is described by considering the diffusion coefficient of Ho(III), because the complex reaction between Ho(III) and organophosphate at the interface is much faster than the reaction on sewage and microtubule [15].

(3) $$J|{}_{t}=\frac{D_{m}^{0}K_{\mathrm{ex}}{\left[{\left(H*R\right)}_{2}\right]}^{3}c_{f}}{d_{m}{\left[H^{+}\right]}^{3}}\left(1-\frac{d_{m}{}^{2}}{6D_{m}^{0}t}\right)$$

Then, *K*_ex_ is constant. *J* signifies the Ho(III) collection flux. *C*_f_ signifies the *Cm* of the wastewater portion. *D*_f signifies_ the collection constant of Ho(III) in microtubes. *d_f_* signifies the thickness of the edge film in the middle of the wastewater portion and microfilter portion. *D*_m_^0^ signifies the Ho(III) constant in microtubes. *d*_m_ represents the thickness of tubes *t* represents time, individually.

### 2.2. Rereagent

The raw wastewaters of a rare earth ore in Northeast China, Arsenazo III, HNO_3_, NaH_2_PO_4_, CH_3_COONa, CH_3_COOH, and Na_2_HPO_4_ are all analytical grade. The organic phosphoric extractant is a commercial collection reagent (purity > 94%). Benzin was distilled at 190–205 °C and filtered by washing with concentrated sulfuric acid.

### 2.3. Preparation of Fluid

This part is also similar to reference [17,18] as below.

For the sake of convenience, the unit mol/L of molar concentration was denoted by “M” in the following research content. 

Ho(III) stock fluid: raw wastewater was dissolved with CH_3_COOH·4H_2_O in 1.2 M nitric acid to configure Ho(III) reserving fluid, and was analyzed that with developer reagent (Arsenazo III).

Ho(III) wastewater fluid: a certain amount of Ho(III) stock fluid quantitatively was diluted to a given molar concentration with CH_3_COONa and CH_3_COOH or Na_2_HPO_4_ and NaH_2_PO_4_, or with 0.10 M nitric acid fluid.

Back collection fluid: various acid solutions were prepared with deionized water.

### 2.4. Test Equipment and Related Measuring Instruments

The devices needed in this study are all self-designed test schemes. The device in this study is an expanded version of the device in reference [17].

The ion *Cm* of the sample containing only trivalent Ho in the feeding fluid was analyzed with a spectrophotometer (Uv-1220) and Arsenazo (III) as the developer (detection wavelength: 652.5 nm).

### 2.5. Experimental Condition Data

For the influence of the current speed of wastewater portion and enriched portion, the speed value in the enrichment portion is from 2.2 to 5.4 mL/min and the speed value in wastewater portion is from 7.4 to 13.2 mL/min.

For the influence of wastewater pH, the pH value is from 3.40 to 5.00.

For the influence of acid *Cm* in enriched portion, acid *Cm*: from 1.0 M to 5.0 M.

For the influence of voluminal proportion of enriched portion (*Vr*), *Vr* is from 0 to 2.0 × 10^−6^ mol/(s·m^2^).

For the influence of Ho(III) *Co* in wastewater portion, *Co* is from 7.00 × 10^−4^ M to 2.00 × 10^−3^ M.

For the influence of organic phosphoric *Cm, Cm* is from 0.060 M to 0.200 M.

For the influence of ion-force in wastewater, ion-force is from 0.5 to 2.0 M.

## 3. Results and Discussion

### 3.1. Constancy of MF-OP

In order to determine the constancy of MF-OP compared with traditional supported fluid film (expressed as SFF), the variation trend of Ho(III) *Cm* in the wastewater portion and enriched portion under the long-standing time static action situation was explored.

In the testing, two running manners were investigated in manner 1: enriched portion traverses the microtube outer shell and wastewater portion traverses the inner shell of the microtube. The current speeds of the wastewater portion and enriched portion are 0.021 m/s and 0.012 m/s, individually. The current speed of the two portions was kept in the testing.

**Figure 3:**
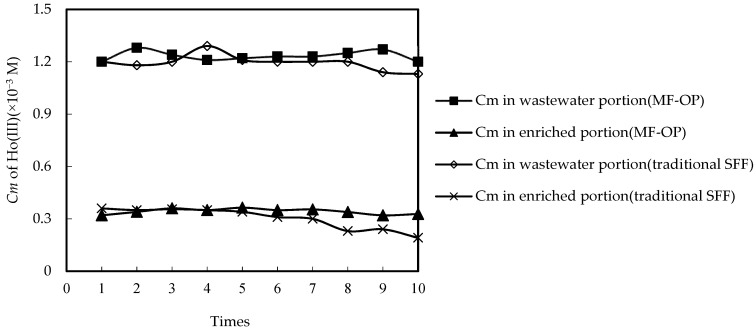
The constancy comparison of MF-OP and traditional SFF.

The nominated theoretical test requirement is to configure the solution pH to 4.60 under the definite wastewater portion pH. *Co* of Ho(III) was 1.25 × 10^−3^ M in the wastewater portion, *Vr* was 0.5, the nitric acid *Cm* of the enriched portion was 3.40 M, and the extractant *Cm* was 0.200 M. The consequences are revealed in Figure 3. We found that after seven times, the change trend of Ho(III) *Cm* was stable. Therefore, we collected two samples in each experiment, once every 100 min and once every 200 min. The Ho(III) *Cm* and steadiness of the enriched portion regularly decline while the traditional supported fluid film is used, and the Ho(III) *Cm* of the wastewater portion and the enriched portion remains stable when MF-OP is used. This is because the organic phosphorus in the traditional supporting fluid film is regularly lost [16,18,19,20,21,22,23], while MF-OP with organic phosphoric fluid may constantly complement the microtube microfilter with organic phosphoric fluid. The results of this study also showed that the stability of MF-OP is indeed better than that of traditional SFF.

### 3.2. Influence of Current Speed of Wastewater Portion and Enriched Portion

**Figure 4:**
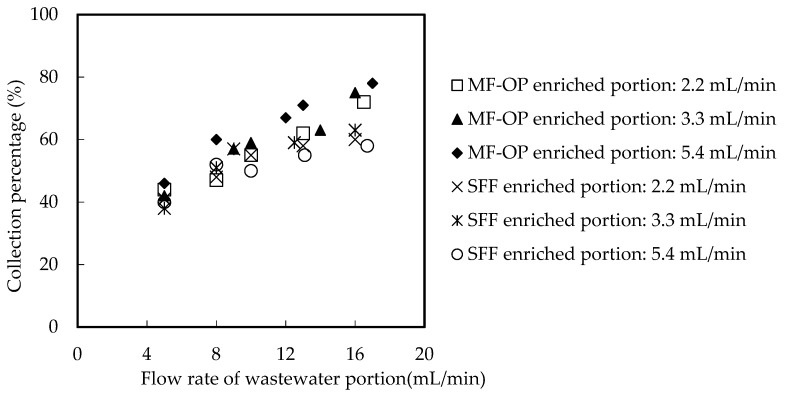
The comparison between MF-OP and traditional SLM for current speeds influence on collection of Ho(III) in both portions (I).

**Figure 5:**
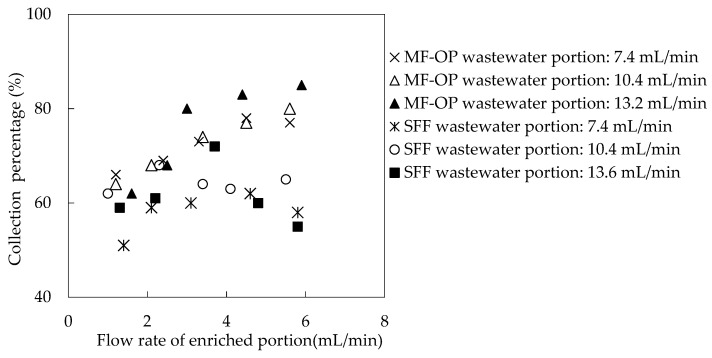
The comparison between MF-OP and traditional SLM for current speeds influence on collection of Ho(III) in both portions (II).

In order to study the collection principle in MF-OP, the main factor controlling the total collection obstruction, it is necessary to consider the fluid dynamics characteristics of the whole system. The current speed of the wastewater and enriched portion plays a vital role in the collection of metallic ion from wastewater back to collection fluid. In the traditional SFF, the extractant will be regularly washed away by the high velocity fluid [16,18]. In MF-OP, organic phosphoric fluid helps to furnish extractant for microtubes under a high velocity fluid situation. Consequently, this portion studies the influence of the current speed of the wastewater portion and enriched portion on the collection rate of Ho(III) [17,24,25,26]. All other parameters, such as pH value, *Co* of Ho(III) in wastewater, *Vr*, and extractant *Cm* were configured to 4.60 and 1.65 × 10^−3^ M, 0.50 and 0.200 M, individually. The influence of the current speed of the wastewater portion and enriched portion on the collection proportion of Ho(III) was revealed in Figure 4 and Figure 5.

We can find that in the traditional supported fluid film (SFF), the high velocity fluid will regularly create the organic phosphoric extractant loss, so the higher the current speed, the lower the collection rate. In case of high-speed liquid flow in the process of MF-OP, organic phosphoric fluid helps to provide a carrier for microtubes. Consequently, the outer wall fluid current speed of the microtube has no significant influence on the collection rate of Ho(III). The current speed of wastewater fluid in the microtube has little influence on the collection rate of Ho(III), because the diffusion rate of wastewater fluid in the inner boundary layer of microtube is a vital rate control step in the whole collection procedure [17,22,23,24,25,26,27]. The current speed of the organic fluid was about 10.4 mL/min in the outer shell of the tube, and the current speed was restricted to 3.3 mL/min.

### 3.3. Influence of Wastewater pH

**Figure 6:**
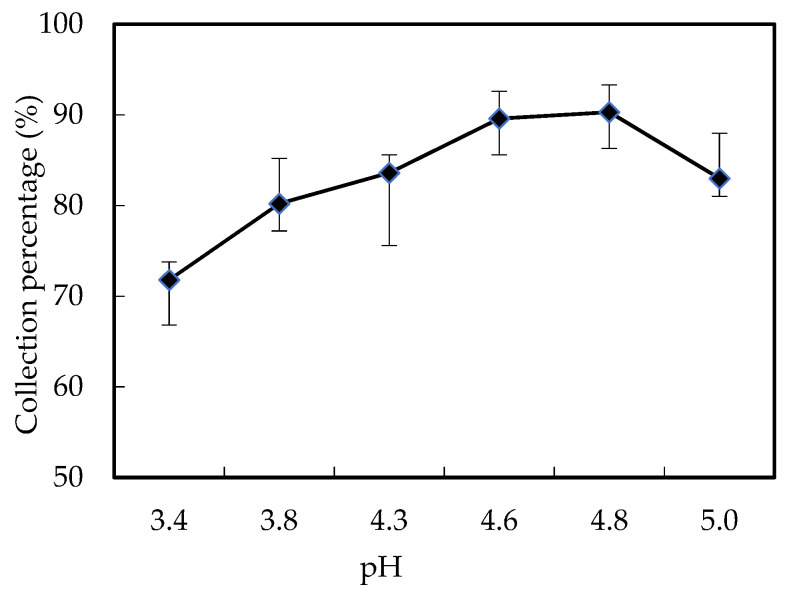
Influence of wastewater pH.

Based on the principle of the collection procedure, the *Cm* difference between the wastewater portion and the enriched portion is the driving force of the mass collection procedure [13]. Thus, in the wastewater portion, the lower the H^+^ *Cm*, the stronger the driving force of the mass collection procedure. Stronger power will promote the collection flux of Ho(III). Similarly, the higher the pH value of wastewater, the higher the collection flux of Ho(III). According to the condition for conventional extraction of the Ho(III), and in order to save the cost of the experiment, we set the pH value range from 3.40 to 5.00. The influence of wastewater pH on Ho(III) collection was studied in the range we set. In the wastewater portion, the *Co* of Ho(III) was 1.68 × 10^−3^ M. In the enriched portion, the *Cm* of nitric acid fluid was 4.00 M, *Vr* was 0.50, and the *Cm* of extractant was 0.180 M. The outcomes are revealed in Figure 6. While the pH value of wastewater raised from 3.40 to 5.00, the collection proportion of Ho(III) raised, and the supreme collection proportion observed at pH 4.60 within 280 min was 87.3%. While the pH value of wastewater is higher than 4.60, the collection proportion of Ho(III) is unsteady in 280 min owing to wastewater emulsification. For all earlier cases, the announcement of relevant documents [13,18] proposed the impact of pH value on the constant of collection procedure. This is on account of the revival influence of organic phosphoric fluid on microtubes and when the dispersion movement of Ho(III) is definitive under stable test state. It can be seen from previous studies that the mass transfer driving force caused by diffusion equilibrium controls the collection process of the system [14,17,18,19,20]. PH value of 4.60 has been chosen as the prime pH in this test.

**Figure 7:**
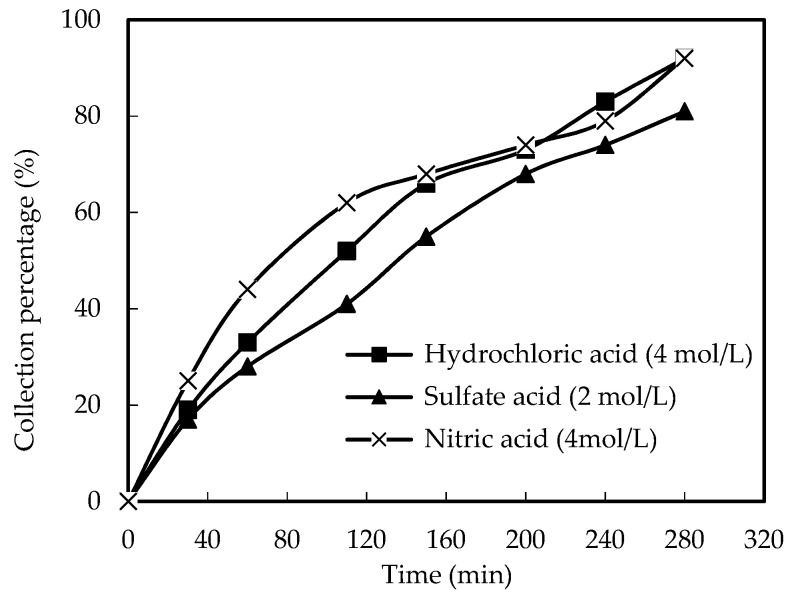
Influence of different acid fluids as resolving reagents.

The strong acid fluid plays a vital role in collecting Ho(III) from wastewater to a resolving stage. Different kinds of acids may also affect the results. Consequently, the influences of distinct acids on the collection of Ho(III) were considered. The pH value of the wastewater portion was adjusted for the *Co* of Ho(III), the *Vr*, and the *Cm* of organic phosphorus to 4.60 and 1.68 × 10^−3^ M, 0.80 and 0.180 M, individually. The influences of different acids in the enriched portion on the collection percentage of Ho(III) are revealed in Figure 7. Under the same acidity situation, sulfuric acid, hydrochloric acid, and nitric acid were applied individually for the resolving reagents. This test found that nitric acid was the most active resolving reagent. According to the research of others, it can be inferred that the effect of sulfuric acid is not good, because the strong oxidation and corrosion of sulfuric acid make the organic solution unstable [19,20].

### 3.4. Influence of Acid Cm in Enriched Portion

**Figure 8:**
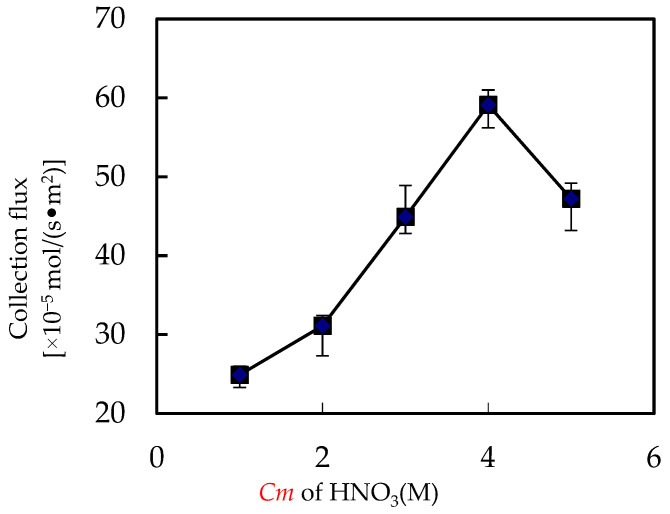
Influence of nitric acid *Cm* on collection of Ho(III).

The resolving reaction of the enriched portion plays a vital role in collecting metallic ion from wastewater to the resolving solution. Consequently, this portion studies the influence of the *Cm* of enriched nitric acid on the collection flux of Ho(III). As for all other parameters, such as pH value, *Co* of Ho(III) in wastewater, *Vr*, and extractant *Cm* were configured to 4.60 and 1.58 × 10^−3^ M, 0.60 and 0.180 M, individually. The influence of nitric acid *Cm* of the enriched portion on the Ho(III) collection flux is revealed in Figure 8. The results showed that the collection flux of Ho(III) raised with the raise of the *Cm* of enriched acid. That can be seen that the efficient acid *Cm* for collection were 3.00 M, 4.00 M, and 5.00 M, individually, which makes the collection flux of Ho(III) 4.35 × 10^−6^ mol/(s·m^2^), 5.89 × 10^−6^ mol/(s·m^2^) and 4.72 × 10^−6^ mol/(s·m^2^).

Increasing the *Cm* of nitric acid fluid from 1.00 M to 2.00 M had no significant influence on the collection flux of Ho(III) for the reason that the quantity of Ho(III) complexes and the *Cm* of organic phosphoric fluid extracted by microtubes per unit time are certain. Since the regulating of acidity and the raise of collection flux, 4.00 M has been chosen as the prime acid *Cm* of the collected portion for the advanced tests.

### 3.5. Influence of Voluminal Proportion of Enriched Portion (Vr)

**Figure 9:**
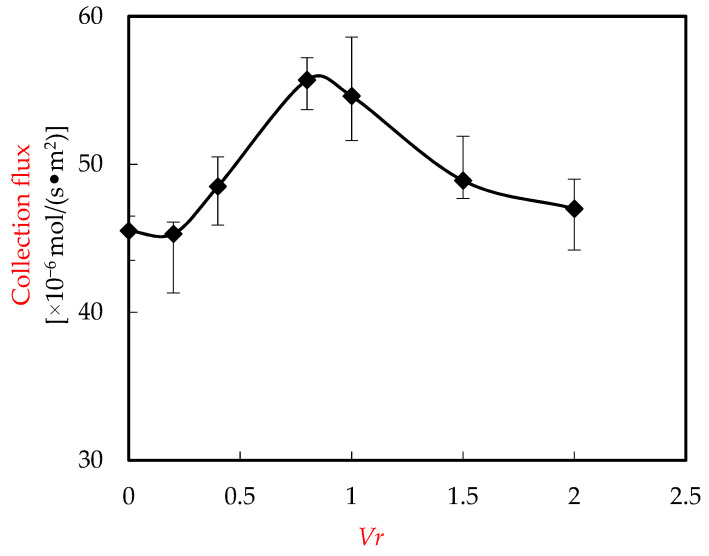
Influence of *Vr*.

The influence of the *Vr* on the collection of Ho(III) was studied. It is assumed that the selected test situation is the specific pH value of the wastewater portion, which is configured to 4.80. The *Co* of Ho(III) in wastewater is configured to 1.58 × 10^−4^ M, the acid *Cm* of the enriched portion was configured to 4.00 mol/L, and the *Cm* of organic phosphoric fluid was configured to 0.120 M. The influence of the *Vr* on the collection of Ho(III) was revealed in Figure 9. The voluminal proportion raised from 0 to 2.00. It can be seen that the most efficient voluminal proportion was 0.60, which made the collection flux of Ho(III) much higher than that of other substances.

This shows that the collection flux of Ho(III) raises with the raise of the voluminal proportion of the enriched portion. While the voluminal proportion of enriched portion raises, the organic phosphoric fluid is obviously dispersed in microtubes. Consequently, the probability of contact between extractant and Ho(III) raises. In this way, the mixing of microtubes and enriched portions provides additional regeneration proportion of the collection surface and microtube surface carrier. Thus, the efficiency of extracting the target heavy metallic-ion complex from organic phosphoric fluid to acid fluid is greatly improved [28,29,30]. When the voluminal proportion raises to a certain extent, the flux decreases due to the reduction of H^+^ in the enriched portion [13,17]. In the subsequent experiment, 0.60 has been chosen for the optimal *Vr*.

### 3.6. Influence of Ho(III) Co in Wastewater Portion

When the *Co* of Ho(III) was in the scope of 8.00 × 10^−5^ M to 2.00 × 10^−4^ M, the influence of *Co* on the collection proportion of Ho(III) was considered. The wastewater portion pH was configured to 4.60, the *Vr* was configured to 0.80, the *Cm* of nitric acid in the enriched portion was also configured to 4.00 M, and the *Cm* of organic phosphorus was 0.20 M. The results are revealed in Table 1. With the Ho(III) *Co* in the wastewater portion from 1.80 × 10^−4^ M raised to 2.00 × 10^−4^ M, the collection proportion of Ho(III) declined. it is that while the interface between the wastewater portion and the microtube is ascertained, the amount of extractant passing through the microtube is certain. That is, in this collection process, the amount of Ho(III) extracted is determined [13,17]. When the Ho(III) *Co* was 7.00 × 10^−4^ M, 1.07 × 10^−3^ M, 1.35 × 10^−3^ M, 1.80 × 10^−3^ M, and 2.00 × 10^−3^ M, and the collection proportions in 180 min were 92.10%, 73.20%, 69.50%, 60.90%, and 48.70%, individually.

### 3.7. Influence of Organic Phosphoric Cm

**Figure 10:**
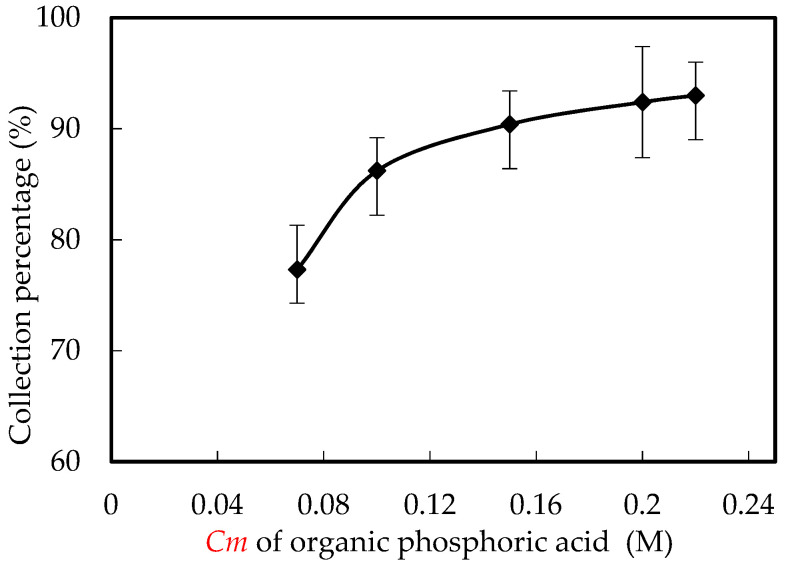
Influence of organic phosphoric *Cm*.

The *Cm* of organic phosphorus in the microtubes and enriched portion also has a vital influence on the collection of Ho(III). The influence of extractant *Cm* on the collection rate of Ho(III) was studied in the range of extractant *Cm* from 0.06 M to 0.220 M. We adjusted the pH value to 4.60, and the *Co* of Ho(III) in the wastewater was 1.80 × 10^−3^ M, the *Vr* of the concentrated portion was configured to 0.80, and the nitric acid *Cm* was also configured to 4.00 M. The results are revealed in Figure 10. The collection rate of Ho(III) raised with the raise of film carrier *Cm* from 0.06 M to 0.220 M. However, when the *Cm* of organic phosphoric fluid raised from 0.200 M to 0.220 M, the collection rate of Ho(III) did not raise significantly. In the range of organic phosphoric *Cm* from 0.060 M to 0.200 M, the availability of organic phosphoric fluid on the fractional microtubes organic interface of wastewater portion raises with the raise of organic phosphoric *Cm*. Consequently, the chemical reaction is balanced to the left. Similarly, when the *Cm* of organic phosphoric fluid becomes low, the equilibrium moves to the right [15,31,32]. When the *Cm* of organic phosphoric fluid raises significantly, the collection flux of Ho(III) will no longer raise with time [13,17,18,29]. When the *Cm* of organic phosphoric fluid was 0.100 M, 0.150 M, 0.200 M and 0.220 M, the collection rates were 85.9%, 89.5%, 93.1% and 93.5% individually. The *Cm* of organic phosphoric fluid is directly proportional to the *Cm* of Ho(III) on microtubes. When the *Cm* of organic phosphoric fluid in the microtube is higher than that of Ho(III) in wastewater, no Ho(III) reacts with excess organic phosphoric fluid, so the raise of Ho(III) collection rate will slow down [17,18]. This shows that when the *Co* of Ho(III) for the action area and time of the film are certain, the amount of extractant extracted by the film per unit time is certain. The best *Cm* of organic phosphoric fluidwas 0.200 mol/L. In this event, the collection proportion of Ho(III) was 93.1% within 280 min.

### 3.8. Influence of Ion-Force in Wastewater

**Figure 11:**
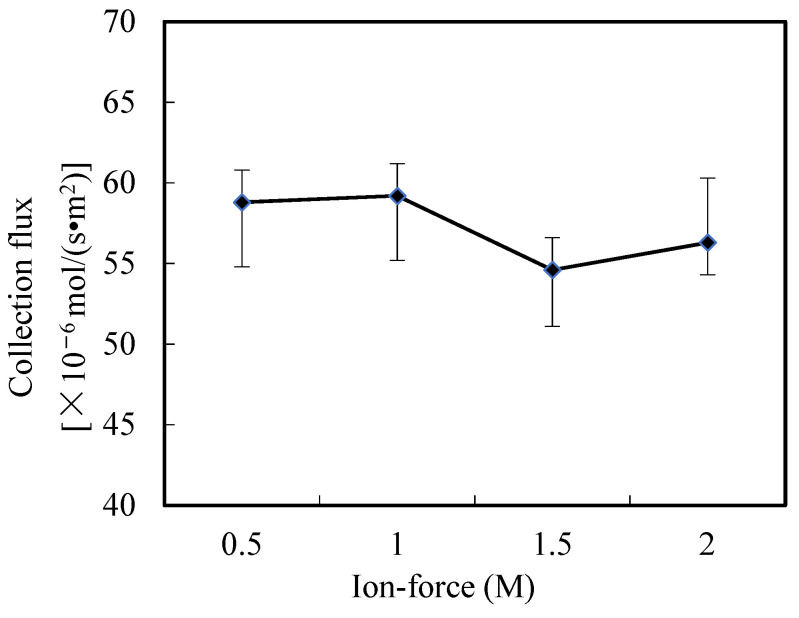
Influence of ion-force on collection of Ho(III) (III).

At this portion, the influence of the ion-force in wastewater on the collection rate of Ho(III) was studied. The results are revealed in Figure 11. The results showed that the ion-force had no significant influence on the collection flux of Ho(III). Because the nitric acid influence surrounded by organic phosphoric fluid is feeble, the ion-force of the enriched portion can be ignored. When the ion *Cm* of the wastewater is low, the ion-force of the two portions can be ignored. Compared with other collection technologies, the situation of action can be easier [17,18,31,32,33,34].

### 3.9. Retention in Microfilter and Resolving Influences

**Figure 12:**
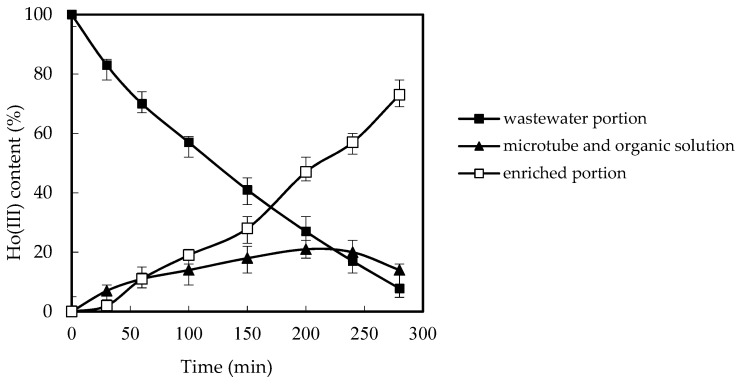
Retention in microtubes and resolving influences.

Under the best situation, the retention in microtubes and resolving influences were considered. We configured the pH to 4.60, and the *Co* of Ho(III) in the wastewater was 1.80 × 10^−3^ M, the *Vr* was configured to 0.80, and the *Cm* of nitric acid fluid in the concentrated portion is also configured to 4.00 M. Additionally, we configured the *Cm* of organic phosphoric fluid to 0.200 M. Based on the *Cm* of Ho(III) in wastewater and resolving stage, the *Cm* of Ho(III) in the PAN microtube can be attained, and then the resolving influence of the enriched portion and the retention phenomenon of the microtube can be obtained. The results are revealed in Figure 12. We can conclude that the retention rate of Ho(III) in the PAN microtubes regularly decreases with the extension of time, because the resolving speed in the collection procedure is faster than the complexation speed.

In previous studies using SLM to remove heavy metals, the retention of heavy metal ions on the membrane was observed [14,15]. In this work, Ho(III) ions have also received attention. The concentration of Ho(III) on the membrane can be calculated according to the concentration of Ho(III) in the wastewater portion and the enrichment portion. The adsorption of Ho(III) on the film increased with the running time. However, with the prolongation of the operation time, the increase rate decreased, which led to a stable Ho(III) percentage on the membrane when the operation time exceeded 200 min.

This is because, as the running time increased, the rate of HoR_3_·3HR in the interface between the membrane and the enrichment portion decreased with the increase in Ho(III) in the enrichment portion of the reaction part. With the increase in operation time, the equilibrium was reached, and the adsorption of Ho(III) was no longer increased. There was about 14% Ho(III) on the membrane surface of the reaction part. However, this has no negative effect on the supply of the membrane. When the experiment was repeated five times, the dislodging rate remained above 80%, which verified the practicability and steadiness of MF-OP. The stability of the membrane and the removal rate of Ho(III) are also enhanced by the separation of the reaction portion from the wastewater portion and enrichment portion in the new design [25,26]. This is the advantage of our newly designed MF-OP, which avoids the carrier shedding that may be caused by the agitator in the traditional SLM method.

### 3.10. Influence of Parameters of Microfilter Microtubes

The influences of microtube structure parameters (inradius of tube, tube–shell thickness, proportion of holes) on mass collection action of Ho(III) collection by MF-OP were also considered. Six kinds of microtubes were carefully chosen, and the following Table 2 lists additional information about these modules.

#### 3.10.1. Influence of Tube–Shell Thickness

**Figure 13:**
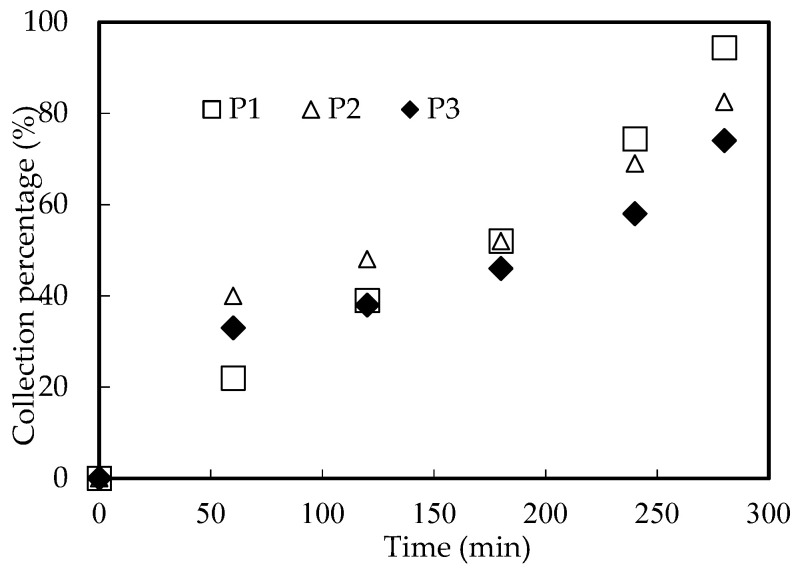
Influence of tube–shell thickness.

The tube–shell thickness will affect the thickness of the organic phosphoric interface layer in the MF-OP module and the collection rate of the Ho(III) complex. Microtubule modules P1, P2, and P3 were selected to study the influence of the tube–shell thickness on Ho(III) collection under the optimal collection situation. The results are revealed in Figure 13. We can conclude that the thicker the tube–shell, the lower the collection rate of Ho(III), because the thickness of the membrane will affect the migration rate of heavy metal ions, carriers, and complexes.

#### 3.10.2. Influence of Tube Holes Proportion

**Figure 14:**
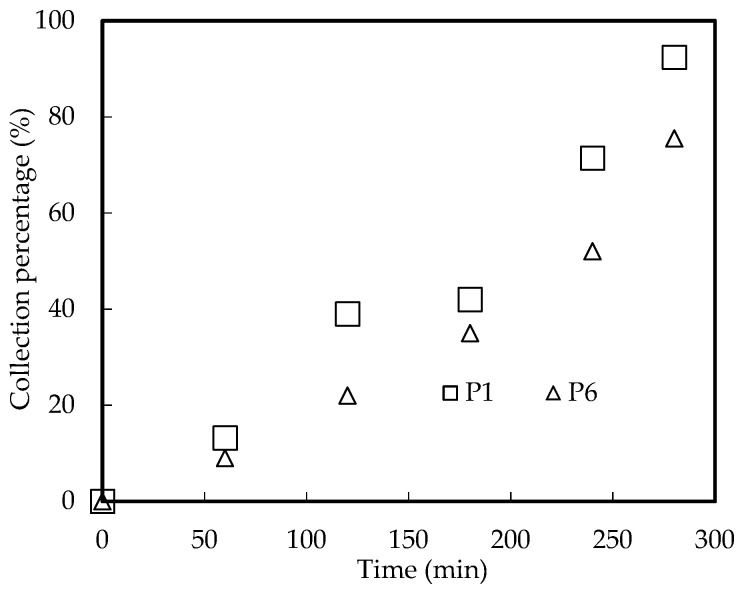
Influence of tube holes’ proportion.

The holes proportion of tubes has a vital influence on the collection proportion of Ho(III) clathrate. Microfilter modules P1 and P6 were carefully chosen to investigate the influence of tube holes’ proportion on the collection of Ho(III) under the optimal collection situation. The results are revealed in Figure 14. We can conclude that the larger the holes’ proportion of tubes, the higher the collection rate of Ho(III). The main reason may be that the larger the holes’ proportion of tubes, the larger the effective collection area of tubes [34].

#### 3.10.3. Influence of Tube Inradius

**Figure 15:**
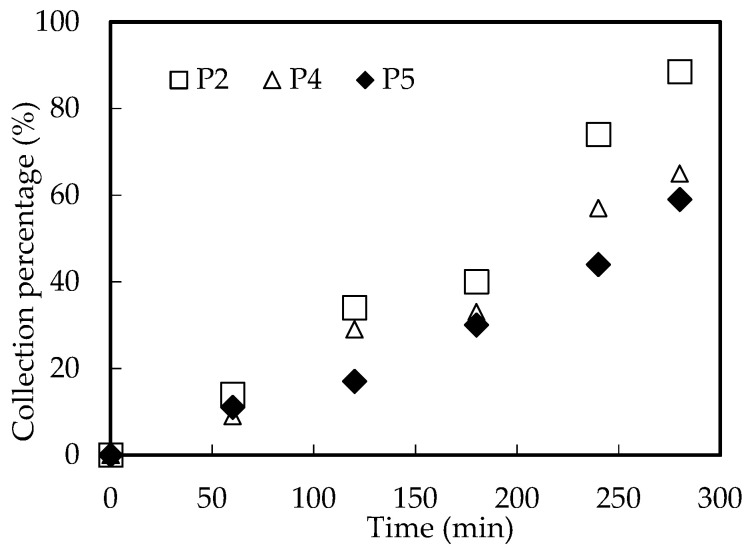
Influence of tube inradius.

The inradius of tube will affect the flow state of fluid and the collection rate of Ho(III) in the MF-OP method. The influence of the tube inradius on the collection of Ho(III) was considered in the best collection situation. The results are revealed in Figure 15. The results showed that the larger the inradius of microtubes, the higher the collection speed of Ho(III). The main reason may be that the bigger the inradius of the tube, the more extractant of the microtube system [16,17,18,35]. Consequently, more organic phosphoric fluid can make the resolving and complexation reaction more efficient.

## 4. Conclusions

It has been found that MF-OP systems using organic phosphoric acid selectively collect Ho(III) in lactic acid medium. We reached the following conclusions: the best collection situation of Ho(III) in the MF-OP system was that the *Cm* of nitric acid fluid was 4.00 M, the *Vr* was 0.80, the *Cm* of organic phosphoric fluid in the enriched portion was 0.220 M, and the wastewater portion pH was 4.60. While *Co* was 1.80 × 10^−3^ M, the collection influence of Ho(III) was very distinct under the prime situation. In the collection time of 280 min, the collection proportion of Ho(III) reached 93.1%.

The constancy of MF-OP was superior compared to traditional supported fluid film (SFF). The outer shell fluid speed of the microtube had little influence on the collection proportion of Ho(III), and the wastewater proportion in tube had little influence on the Ho(III) collection proportion. As time goes on, the retention proportion of Ho(III) in microtubes declined regularly. The collection proportion of Ho(III) raised with the decline in tube thickness, the raise in microtube inradius, and the increase in microtube holes’ proportion. This study tried to discover the method system of Ho(III) extraction.

At present, the research was still in the experimental stage in the laboratory. After the experimental research, this technology may be applied in practice. The selected optimal operation conditions can break through the bottleneck of water treatment and sheeting technology. Future research needs to focus on various materials of the device, cost reduction, and optimization of the process for pilot study.

We can apply the collected Ho(III) to the alloy industry, photoelectricity industry, and medicine manufacturing. It can make great contributions to the country’s industrial development. Of course, this requires the joint efforts of our various industries, good national policies, cost reduction, and the establishment of a good bridge for Ho(III) recycling. It can also provide scientific and theoretical support for the treatment of industrial waste residue, especially the purification and extraction of heavy metals.

## Figures and Tables

**Table 1:** Influence of Ho(III) *Co*.

Time (Min)	Collection Proportion (%)				
	7.00 × 10^−4^ M	1.07 × 10^−3^ M	1.35 × 10^−3^ M	1.80 × 10^−3^ M	2.00 × 10^−3^ M
0	0	0	0	0	0
60	40.70	39.70	27.20	22.70	14.30
120	81.20	54.60	42.90	36.70	21.40
180	92.10	73.20	69.50	60.90	48.70
240	-	87.30	80.10	77.90	61.20
280	-	-	89.30	90.10	71.40

**Table 2:** Parameters of microtube module construction.

No	Parameters of Microtube Construction				
	Length of Tube, *L*/(m)	Proportion of Holes	Number of Tubes	Inradius of Tube, *d*_i_/(mm)	Thickness of Tube–Shell, *d*_m_/(mm)
P1	0.30	63%	28	2.93	0.31
P2	0.30	63%	34	2.93	0.54
P3	0.30	63%	28	2.93	0.62
P4	0.30	63%	34	2.22	0.53
P5	0.30	63%	28	1.71	0.44
P6	0.30	22%	36	2.93	0.31

## Data Availability

Not applicable.
